# Sixty years of the first studies by Horácio Martins Canelas on Wilson's disease

**DOI:** 10.1055/s-0045-1802553

**Published:** 2025-02-24

**Authors:** Egberto Reis Barbosa, Jacy Bezerra Parmera, Rubens Gisbert Cury, Eduardo Luiz Rachid Cançado, Patrícia Áurea Andreucci Martins Bonilha, Hélio Afonso Ghizoni Teive

**Affiliations:** 1 Universidade de São Paulo, Faculdade de Medicina, Departamento de Neurologia, São Paulo SP, Brazil.; 2 Universidade Federal do Paraná, Faculdade de Medicina, Departamento de Medicina Interna, Curitiba PR, Brazil.; 3 Universidade de São Paulo, Faculdade de Medicina, Departamento de Gastroenterologia, São Paulo SP, Brazil.

**Keywords:** Hepatolenticular Degeneration, Kayser-Fleischer Rings, Fibrosis, History

## Abstract

Research into Wilson's disease (WD) in Brazil had the effective participation of Professor Horácio Martins Canelas, from the Neurologic Clinic of the Teaching Hospital of the School of Medicine of Universidade de São Paulo (USP). His exponential contributions to the study of WD placed Brazil on the international stage, making USP's Neurologic Clinic one of the world's leading research centers in the area of neurodegenerative diseases with metal accumulation.

## INTRODUCTION

Wilson's disease (WD) is an autosomal recessive disorder of the copper metabolism, leading to its accumulation in different organs and tissues. Hepatic and neurological symptoms are its main clinical features. 1 2

Prior to Kinnier Wilson's classic description, WD was described by various authors in the form of case reports, such as Frerichs in 1861, Westphal in 1883 (“Pseudosclerosis”), Gowers in 1888 (“Tetanoid chorea”) and in 1906 (“On tetanoid chorea and its association with cirrhosis of the liver”), Strümpell in 1898 and 1899, Omerod in 1890, and Homén in 1890. 1 2 3

In 1902, Kayser 4 reported the presence of a greenish-brown ring around the cornea of a 23-year-old man, thought to have multiple sclerosis. The following year, Fleischer 5 described the same pigmented ring in a case of “pseudosclerosis” and in another case of alleged multiple sclerosis.

In 1902 and 1903, Kayser 4 5 reported the presence of a greenish-brown ring around the cornea of two patients, who were thought to have multiple sclerosis, with one of them also being reported with “pseudosclerosis.”

In 1911, Wilson presented his monograph describing the “progressive lenticular degeneration”, which resulted in the publication of a historical paper in *Brain Journal* , with the title “Progressive lenticular degeneration: a familial nervous disease associated with cirrhosis of the liver”, the following year. 1 In these seminal reports, Wilson described four personal cases (three of them with neuropathological study), two referred by Gowers and Ormerod, and six other cases from the literature. He emphasized the familial character of some cases, as well as the presence of cirrhosis of the liver, mostly asymptomatic. However, Wilson believed the liver did not contribute to the disease's clinical progression. 1

Wilson did not note, in his first papers, a relationship between his cases and the pseudosclerosis of Westphal-Strümpell. However, 2 years later, while writing on “progressive lenticular degeneration,” the author mentioned similarities between the entities. 6

After Wilson's seminal publication, research into the disease took a major step forward, with evidence of an increase in the amount of copper in the liver, as well as evidence of an elevation in copper concentration in the urine of the patients with WD. 6 In 1948, Cumings 7 proved the accumulation of copper in the liver and brain of this cohort.

Treatment plans with copper chelating agents were unsuccessful until the introduction of the penicillamine by Walshe, 8 in 1956, which made long-term treatment of the disease possible. Until then, the disease had been invariably fatal.

## CANELAS' CONTRIBUTION TO THE STUDY OF WILSON'S DISEASE IN BRAZIL

The Brazilian neurological literature registers one WD case report made by Austregésilo Filho, 9 in 1944. The second case registered was a 20-year-old patient studied in Division of Neurology of the Teaching Hospital of the School of Medicine of Universidade de São Paulo (USP), under the supervision of Drs. Ibrahim Mathias and Oswaldo Lange, with the typical “wing beat tremor” and Kayser-Fleischer's rings. 10

**Figure 1:**
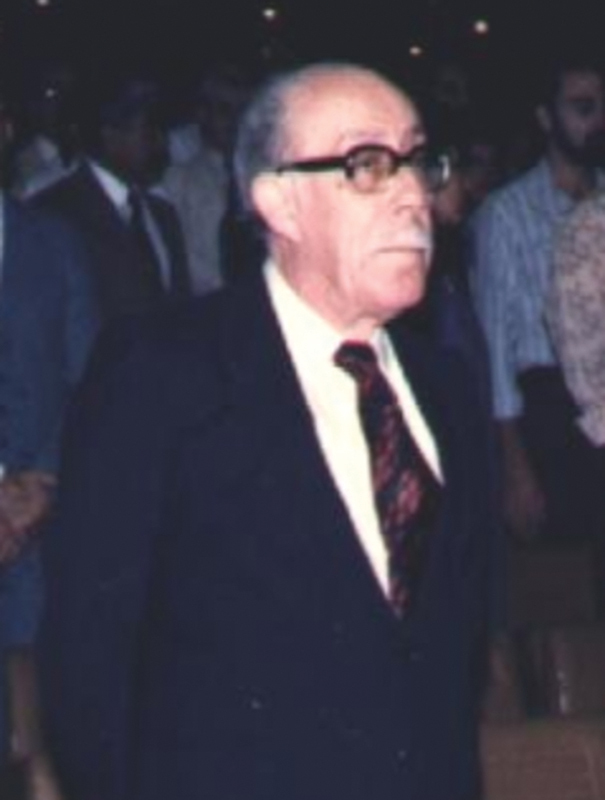
Professor Horácio Martins Canelas (1919–1995) at the Brazilian Neurology Congress, Belo Horizonte, 1986.

At the beginning of the 1960s, penicillamine had already been used in several countries in Europe and the USA and its efficacy was definitively proven. Thus, WD left a long list of untreatable, inherited, metabolic neurological diseases and begun to attract the interest of a growing number of researchers worldwide, focused on precise diagnosis and correct treatments. 6 8 At that time, in Brazil, Horácio Martins Canelas ( Figure 1 ) developed a project to investigate WD at USP's Neurologic Clinic, which would become one of the most fruitful lines of research in the following decades. Horacio Martins Canelas was a full professor of Clinical Neurology at USP's School of Medicine. He was a disciple of Professor Aderbal Tolosa, who replaced Professor Enjolras Vampre, a pioneer of neurology in the state of São Paulo who died prematurely as a full professor at USP's School of Medicine.

To develop this research project, Canelas collaborated with Francisco Bastos de Jorge, who cooperated with laboratorial support. As such, in 1962, Canelas published, along with de Jorge and Costa-Silva, a paper focusing on the copper determination methodology in biological materials. 11 In the following year, Canelas published, with de Jorge and Spina-França's participation, a study concerning the average values of copper in the blood, cerebrospinal fluid, and urine. 12 In 1964, again with de Jorge, a study of ceruloplasmin levels in normal individuals was published. 13 In the same year, Canelas and his collaborators achieved an international publication in *Clinica Chimica Acta* (Amsterdam, Netherlands) concerning copper concentration in the saliva, salivary glands, and pancreas. 14 With these pioneering studies, Canelas and his team achieved the know-how of laboratorial tools to evaluate copper metabolism.

Besides the laboratorial investigations, Canelas and his team reported important clinical studies. 15 16 Thus, in 1963, Canelas and his collaborators published a detailed study of 3 cases of WD, two of which included neuropathological examination. 15

In 1967, Canelas, de Jorge, and Tognola published a fascinating comparative study of copper metabolism in patients with WD undergoing a vegetarian and mixed diet in the *Journal of Neurology, Neurosurgery, and Psychiatry* . 17

Canelas' main publications on WD are summarized in Table 1 .

**Table 1:** Canelas' main publications on Wilson's disease

Year	Journal	Topic
1963	*Arq Neuropsiquiatr*	Ceruloplamin levels in the blood
1963	*Rev Paul Med*	Copper metabolism – blood, CSF, and urine
1963	*Arq Neuropsiquiatr*	WD – Clinical and biochemical study
1964	*Clin Chim Acta*	Copper metabolism – saliva, salivary glands, and pancreas
1966	*Rev Paul Med*	Case series of WD
1966	*Rev Bras Oftalmol*	Copper levels in ocular tissues
1967	*J Neurol Neurosurg Psychiatr*	Metabolic balances of copper and diets
1976	*Rev Hosp Clin Fac Med São Paulo*	WD and pregnancy
1976	*Arq Neuropsiquiatr*	Copper and ceruloplasmin blood serum of peripheral veins and pre-hepatic veins
1978	*Acta Neurol Scand*	WD and osteoarhtopathy
1978	*Acta Neurol Scand*	WD and heart involvement
1985	*Arq Neuropsiquiatr*	WD – study of 95 cases
1987	*Arq Neuropsiquiatr*	WD abdominal ultrasonography – 33 patients
1987	*Arq Neuropsiquiatr*	WD – 102 cases
1990	*Arq Neuropsiquiatr*	WD and non-Wilsonian extrapyramidal syndrome – case report
1991	*Arq Neuropsiquiatr*	Case series – 76 WD treated patients
1992	*Arq Neuropsiquiatr*	WD and Zinc treatment – report of 3 cases
1993	*Rev Paul Med*	WD – Brain MRI and clinical correlation in 16 patients

Abbreviations: *Acta Neurol Scand* , *Acta Neurologica Scandinavica* ; *Arq Neuropsiquiatr* , *Arquivos de Neuro-Psiquiatria* ; *Clin Chim Acta* , *Clinical Chimica Acta* ; CSF, cerebrospinal fluid; *J Neurol Neurosurg Psychiatry* , *Journal of Neurology, Neurosurgery and Psychiatry* ; MRI, magnetic resonance inaging; *Rev Bras Oftalm* , *Revista Brasileira de Oftalmologia* ; Rev Hosp Clin Fac Med São Paulo, Revista do Hospital de Clínicas da Faculdade de Medicina da Universidade de São Paulo; *Rev Paul Med* , *Revista Paulista de Medicina* ; WD, Wilson's disease.

With these publications, Canelas raises USP's Neurologic Clinicas a national and international reference center for WD research. This line of investigation remains one of the most traditional of USP's Neurologic Clinic and, for the past 36 years, has counted with an effective participation of researchers from the Gastroenterology Department. 18 19

Professor Canelas was my advisor (ERB) in my master's and doctorate theses, both of which had WD as their theme. In the preparation of these academic works, through his constant and dedicated collaboration, I was able to observe his talent as a university professor, capable of transmitting the complex guidelines for the elaboration of a scientific text in a didactic manner, with the strictest standards. Professor Canelas was deeply knowledgeable about Brazilian Portuguese, and everyone who had the privilege of writing academic texts under his guidance improved the quality of their scientific writing.
